# High-Intensity Training Telerehabilitation for Persons with Chronic Low Back Pain: A Pilot Clinical Trial

**DOI:** 10.3390/jcm13247599

**Published:** 2024-12-13

**Authors:** Timo Meus, Annick Timmermans, Sim Klaps, Jonas Verbrugghe

**Affiliations:** 1 REVAL Rehabilitation Research Center, University of Hasselt, 3500 Hasselt, Belgium; annick.timmermans@uhasselt.be (A.T.); sim.klaps@uhasselt.be (S.K.); jonas.verbrugghe@uhasselt.be (J.V.); 2 MOVANT Research Group, Department of Rehabilitation Sciences and Physiotherapy (REVAKI), University of Antwerp, 2610 Antwerp, Belgium

**Keywords:** chronic nonspecific low back pain, high-intensity training, telerehabilitation, blended care

## Abstract

**Background/Objectives:** High-intensity training (HIT) has been shown to enhance physical fitness and reduce functional impairments in persons with moderately disabling chronic nonspecific low back pain (CNSLBP). However, sustaining these improvements post-rehabilitation remains a challenge. To address this, a home-based, technology-supported HIT program utilizing telerehabilitation can be implemented at home. This study assesses the feasibility and clinical effectiveness of a telerehabilitation HIT program for persons with CNSLBP. **Methods**: The pilot clinical trial (NCT05234008) recruited 15 persons with CNSLBP. Participants completed a 6-week multimodal HIT intervention with 12 bi-weekly sessions. The first four sessions were organized at REVAL Research Center, followed by eight home-based sessions using the Physitrack^®^ platform. Assessments were conducted at baseline (PRE), two weeks into the intervention (MID), and immediately post-intervention (POST). Outcome measures included maximal oxygen uptake (VO2max) testing, disease-related outcomes, feasibility, motivation assessed via questionnaires, and system usability and adherence tracked through Physitrack^®^ technology. **Results**: Fourteen participants (seven females; age: 45.9 years) successfully completed the program without adverse events. Based on PRE–POST comparisons, motivation levels remained high (Motivation Visual Analog Scale: −1.2 ± 0.9, *p* = 0.043) despite reduced motivation at POST. Improvements were also observed in pain (Numeric Pain Rating Scale: −1.8 ± 0.2, *p* = 0.026), disability (Modified Oswestry Disability Index: −12.1 ± 10.2, *p* = 0.002), fear-avoidance (Fear-Avoidance Components Scale: −10.1 ± 5.8, *p* = 0.005), and exercise capacity (VO2max: 4.4 ± 1.6, *p* = 0.048). **Conclusions**: The HITHOME study is the first to investigate the feasibility and effectiveness of a telerehabilitation HIT program for persons with CNSLBP. The results underscore the feasibility of implementing a home-based HIT program to support adherence to vigorous exercise programs and improve clinical outcomes in this population. Additionally, the findings emphasize technology’s potential importance in enhancing home-based exercise therapy and lay the groundwork for future studies on blended care and telerehabilitation using HIT in CNSLBP.

## 1. Introduction

Chronic nonspecific low back pain (CNSLBP) represents the leading cause of disability among musculoskeletal disorders globally, significantly contributing to disability rates [1,2,3]. Predictions indicate a sharp rise in the prevalence of CNSLBP in the coming decades, highlighting the urgent need for effective intervention strategies [4]. This challenge is particularly important because, without a targeted effort to understand and address CNSLBP, it will likely further strain healthcare resources, diminish quality of life, and result in considerable economic costs on a global scale [5].

Exercise therapy has consistently proven to be effective in improving clinical outcomes in persons with CNSLBP. However, the overall effect size remains moderate. This is possibly attributable to the differentiating trials describing these results, wherein exercise intensity, duration, and training methods are inconsistently applied, and pooled data become constrained by the considerable heterogeneity in study design. As a result, the ability to accurately assess the relative impact of these variables on therapeutic outcomes is complicated [6]. High-intensity training (HIT) has emerged as a particularly promising intervention. Recent evidence highlights both the feasibility and therapeutic benefits of HIT for a broad range of populations, including healthy adults as well as persons managing chronic health conditions such as cardiovascular disease and metabolic syndrome [7,8,9,10]. Furthermore, multiple studies demonstrate that the benefits of HIT extend beyond healthy adults and those with chronic health conditions, also resulting in significant improvements in physical fitness and reductions in disability compared to moderate-intensity training in persons with CNSLBP [11,12,13]. However, a major challenge remains sustaining adherence to exercise regimens after discharge from supervised care. As such, discontinuing exercise often leads to the loss of gains achieved during the intervention [11,14,15].

To mitigate the issue of lost gains, telerehabilitation programs have increasingly been recognized as a viable option for managing persons with musculoskeletal disorders (including persons with CNSLBP) [16]. They support benefits such as improved pain and function, high adherence to therapy, and improved cost-effectiveness compared to conventional therapies [16]. However, telerehabilitation programs also pose several challenges, such as the quality of exercise performance, accurate supervision, and the inadequate use of structured exercise regimens [17,18].

A potential solution to these challenges in musculoskeletal disorders, including CNSLBP, is the integration of HIT through telerehabilitation [19]. In addition to improved clinical efficacy [12,13], combining HIT with telerehabilitation has the potential to enhance patient satisfaction, motivation, and adherence to home exercise programs, with lower drop-out rates than conventional therapy [20]. Moreover, it provides the opportunity to provide structured guidance and monitoring, facilitating the preservation of therapeutic gains achieved and providing sustained motivation and adherence in the long term, further supporting the effectiveness of telerehabilitation programs [21,22].

The pilot clinical trial aimed to evaluate the feasibility and clinical effectiveness of a telerehabilitation HIT program for persons with CNSLBP. The primary objectives were as follows: (1) to assess the feasibility of performing HIT at home in persons with CNSLBP and (2) to evaluate the feasibility of using Physitrack as a supportive technology during home-based HIT. The secondary objective was to estimate and describe the potential changes in clinical outcomes for participants with CNSLBP performing a HIT telerehabilitation program.

## 2. Materials and Methods

This clinical pilot trial, referred to by the acronym ‘HITHOME’ as registered, was preregistered (ClinicalTrials.gov identifier: NCT05234008) and adhered to the extension of the Consolidated Standards of Reporting Trials (CONSORT) statements for pilot and feasibility trials [23].

### 2.1. Trial Design

**Figure 1:**
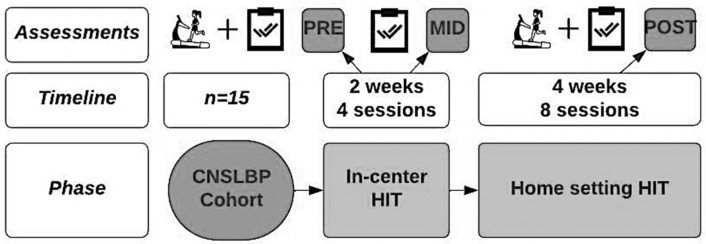
Study design.

A pilot clinical trial was conducted with a single group of participants diagnosed with CNSLBP. As part of the study, these participants underwent a six-week HIT exercise intervention program consisting of 12 rehabilitation sessions (2 per week). The initial four training sessions were provided in-center at REVAL to ensure a standardized base and solid foundation for participants. These in-center sessions allowed for proper instruction, feedback on execution, and progression of the exercises, ensuring that participants were performing the exercises correctly and safely before transitioning to the telerehabilitation HIT program. The subsequent eight sessions were conducted in each participant’s home, supported by the Physitrack mobile application (Physitrack, London, UK, https://www.physitrack.com, accessed from January 2023 until December 2024) for participants and a software platform for researchers. An overview of the study design is shown in Figure 1.

### 2.2. Participants

Participants were screened and recruited regionally in Limburg, Belgium, through local advertisements and social media (i.e., flyers) between January 2023 and December 2024 (end of study).

### 2.3. Eligibility Criteria

The inclusion criteria for participants were as follows: (1) Participants were restricted to Dutch-speaking individuals to ensure they could fully engage with the study protocol and educational materials, which were specifically designed in Dutch. This criterion was essential to guarantee participants’ thorough understanding of the content, thereby supporting the feasibility and overall integrity of the study, (2) aged between 25 and 60 years, (3) diagnosed with chronic low back pain of nonspecific origin by a physician, defined as pain localized below the costal margin and above the inferior gluteal folds, with or without referred leg pain of a nociceptive mechanical nature, persisting for at least 12 weeks, and not attributable to any identifiable specific pathology [24] (i.e., infection, tumor, osteoporosis, fracture, structural deformity, inflammatory disorders, radicular syndrome, or cauda equina syndrome). Participants were excluded if they (1) had a history of spinal fusion surgery, (2) had any form of cardiac disease, (3) were diagnosed with any acute or chronic musculoskeletal disorder other than CLBP that could interfere with the proper execution of the therapeutic program, (4) had comorbidities (i.e., paresis and/or sensory disturbances due to neurological conditions, diabetes mellitus, rheumatoid arthritis), or (5) had ongoing compensation claims and/or work disability lasting more than six months.

### 2.4. Recruitment Strategy

Potential participants who contacted the researchers and met the preliminary inclusion criteria outlined in the flyer were informed about the study details by one of the researchers via telephone or email. If they remained interested, the study protocol, along with the information and consent form, was provided to them either as a hard copy or via email, depending on their preference. The researchers followed up with the potential participants within seven days to address any final questions and confirm their willingness to participate. Subsequently, the participants signed and returned the consent form for final enrollment within two weeks.

### 2.5. Intervention

The intervention consisted of one in-center phase (two weeks, four sessions) and one at-home phase (four weeks, eight sessions).

#### 2.5.1. In-Center Phase

Participants performed a high-intensity exercise protocol lasting 1–1.5 h at REVAL Research Center, following a protocol previously established and published by our research group, which includes high-intensity cardiorespiratory, general resistance, and core strength training [12].

Cardiorespiratory training was structured around an interval protocol on a cycle ergometer, consisting of five high-intensity, one-minute bouts performed at 110 revolutions per minute (RPM), corresponding to 100% of the VO2max workload determined during the maximal cardiopulmonary exercise test. Each bout was followed by one minute of active recovery at 75 RPM, set at 50% of the VO2max workload. The recovery interval remained constant throughout all sessions.

General resistance training involved six exercises targeting both the upper and lower body, performed on fitness machines. A one-repetition maximum (1RM) test was conducted for each exercise to establish the maximal weight capacity. During the initial session, participants performed one set of up to twelve repetitions at 80% of their determined 1RM for each exercise. The load was progressively increased when participants were able to complete more than ten repetitions across two consecutive training sessions, ensuring an adaptive increase in resistance and muscle development.

Core strength training comprised six static core exercises selected for their ability to activate the core muscles at an intensity of no less than 40–60% of the maximum voluntary contraction. Each exercise consisted of one set of ten repetitions, with each repetition involving a ten-second static hold. Participants were instructed to sustain the final repetition for as long as possible. The intensity of these exercises was progressively increased by extending the static hold duration or advancing to more demanding postures once the participant consistently maintained a stable core position for the prescribed duration over two consecutive sessions.

Physitrack technology use during the in-center phase: Participants were already instructed to download the Physitrack mobile application on their phones (Android/Apple). This GDPR and HIPPAA-compliant, cloud-based digital platform was free for the participants and allowed health professionals to assign exercises and programs (with training dosage) to participants remotely, track progress, provide feedback in real-time, and send reminders. The researcher checked whether the app worked correctly and provided information on how to use it (which is necessary for the next phase).

#### 2.5.2. Home Phase

Following the in-center phase, participants performed eight sessions, conducted twice per week over four weeks, consisting of high-intensity cardiorespiratory and core muscle training utilizing the same principles of progression and regression employed during the in-center phase.

**Figure 2:**
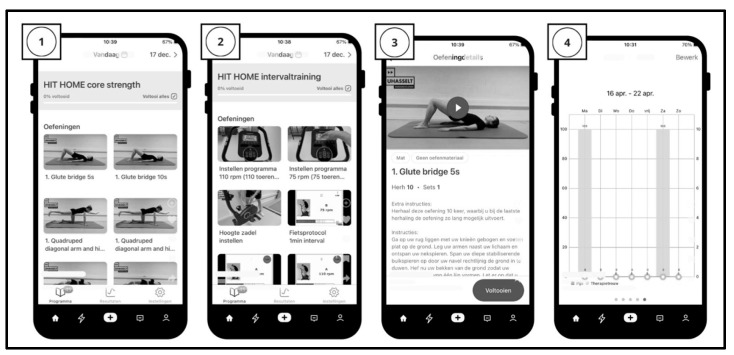
Visualization of the HIT program in the Physitrack mobile application consisting of trunk strength exercises (1) and cardiorespiratory interval training (2). Exercises were displayed with a video/picture guide and written instructions (3). The therapist could follow the participant’s adherence through a clear progression graph (4).

Cardiorespiratory and core strength training: During eight home sessions, participants were provided with a fitness bike, a smartwatch (Polar M200), a training mat, and resistance bands for cardiorespiratory and core strength training. Using the Physitrack program (Figure 2), researchers designed a personalized telerehabilitation HIT program for each participant to perform on the bike and training mat. The HIT program closely resembled the in-center phase and lasted approximately one hour each session.

The Physitrack system enabled automated reminders for exercise sessions and recorded exercise completion, including sets, repetitions, and rate of perceived exertion (RPE) for each exercise. It also allowed for real-time feedback and messaging between participants and researchers for monitoring and review. Each exercise had a specific prescribed training dose (frequency, sets, and repetitions), and participants were asked to report their RPE using the app’s 10-point Likert scale.

Researchers reviewed and adjusted each participant’s program weekly (every two sessions) as needed, based on the self-reported RPE and exercise completion data from the Physitrack platform. They also checked the system daily for urgent alerts or messages from participants. The smartwatch recorded heart rate during the cardiorespiratory interval training, and participants entered their training data into the Physitrack platform.

### 2.6. Outcome Measures

Participants underwent assessments at three time points: baseline (PRE), after two weeks of in-center rehabilitation (MID), and at the end of the home sessions (POST). PRE and POST assessments were conducted at the REVAL Research Center, Hasselt University, while MID assessments were conducted online via a Qualtrics survey. During PRE-assessment, questionnaires were discussed in advance with the participant by the researcher and were completed by the participant solely in a quiet room. When starting the MID assessment, participants were guided through a start-up webpage that provided the same information regarding the questionnaires. The PRE and POST assessments lasted approximately 75 min (30 min for physical testing and 45 min for questionnaires). During the PRE assessment, the following sociodemographic data were collected before beginning the measurements: BMI, gender, age, education, profession, family and work situation, and lifestyle factors (i.e., diet and smoking). The MID assessment lasted around 45 min and consisted solely of questionnaires. Participants received an email from the researchers with a personalized weblink, directing them to the online survey for the MID assessment.

#### 2.6.1. Feasibility Measures

The nominal Motivation Visual Analog (MVAS) and Satisfaction Visual Analog Scales (SVAS) were used to evaluate motivation for rehabilitation and satisfaction with rehabilitation and consists of a line indicating eleven successive scores (0–10), whereby zero means ‘no motivation/satisfaction’ and ten means ‘very high motivation/satisfaction’.

Intrinsic motivation for the technology-supported HIT was assessed by the Intrinsic motivation inventory (IMI). This is a nominal 35-item questionnaire that assesses the multidimensional subjective experience while performing a certain activity, yielding six subscales (interest/enjoyment, perceived competence, effort, value/usefulness, felt pressure and tension, and perceived choice), with the possibility of independent scoring for each scale and a general scoring. A higher score correlates to higher intrinsic motivation (total range 35–245) [25].

The System Usability Scale (SUS) assesses the perceived usability of Physitrack. The SUS is a standard 10-item questionnaire in which responses are measured on a 5-point Likert scale ranging from 1 (strongly disagree) to 5 (strongly agree). A total SUS score yields scores ranging between 0 (worst) and 100 (absolute best). A score > 68 is considered above-average usability, and >80 is considered high usability and a level at which participants are likely to recommend the product to peers [26].

Therapy adherence to the exercise program was evaluated by counting the number of completed therapy sessions within the six-week protocol. Therapy adherence (i.e., the number of sessions completed, number of exercises, and sets and repetitions completed (all expressed as a percentage) within each session) were recorded within the Physitrack system. The program was considered feasible if at least 90% of the participants completed the trial and the adherence to the program was at least 75% (equivalent to at least 6 out of 8 sessions in total performed) [27].

Adverse events were recorded by asking the participants to record any adverse events directly into the Physitrack App so researchers could review them. An adverse event was defined as an intervention-related event resulting in the absence of or modification to the exercise intervention.

#### 2.6.2. Clinical Effectiveness Measures

The Brief Pain Inventory short form (BPI-sf) evaluates the severity of a patient’s pain and the impact of this pain on the patient’s daily functioning. The BPI-sf consists of 9 items on a 10-point scale. The patient is asked to rate the worst, lowest, mean, and current pain intensity, list current treatments and their perceived effectiveness, and judge the degree to which pain interferes with general activity, mood, walking ability, normal work, relationships with other persons, sleep, and quality of life. This questionnaire is reliable and valid for use in persons with chronic low back pain [28].

The Modified Oswestry Disability Index (MODI) evaluates the limitations persons experience in their daily activities due to chronic low back pain. The MODI consists of 10 items that can be scored on a 5-point scale. The patient’s restriction percentage can be indicated based on the total score. This questionnaire is reliable and valid for use in persons with chronic low back pain [29].

The International Physical Activity Questionnaire—short form (IPAQ-sf) estimates physical activity levels. The IPAQ-sf consists of 7 questions. A higher score corresponds to a more physically demanding activity level. This questionnaire is reliable and valid for use in persons with chronic low back pain [30].

The Fear-Avoidance Components Scale (FACS) evaluates fear-avoidance in patients with painful medical conditions and includes constructs such as pain-related catastrophic cognitions, hypervigilance, and avoidance behaviors. The FACS consists of 20 items with a score from 0 (totally disagree) to 5 (totally agree), with a total possible score of 100. The following anxiety avoidance severity levels are recommended for clinical interpretation: subclinical (0–20), mild (21–40), moderate (41–60), severe (61–80), and extreme (81–100) [31].

#### 2.6.3. Physical Assessment

For the maximum exercise test, a bicycle ergometer (eBike Basic, General Electric GmbH, Bitz, Germany) with pulmonary gas exchange analysis (MetaMax 3B, Cortex Biophysik GmbH, Leipzig, Germany) was used. Oxygen uptake (VO2max), expiratory volume (VE), and respiratory exchange rate (RER) were tracked every breath, and an average will be taken every 10 s. Heart rate was continuously monitored using a heart rate chest strap (H10, Polar Electro Inc., Kempele, Finland). Weight and height were measured with a precision-calibrated weighing scale and height meter. After a five-minute warm-up, a step-by-step resistance protocol (80 reps/minute, starting at 40 Watts, increasing with 20 Watts every minute) was used until the maximum wattage was reached (=no longer able to maintain a stable 80 revolutions per minute) [32,33]. This assessment was used to assess changes in cardiorespiratory fitness and establish the baseline VO2max, indicating that improvements in VO2max represent increased cardiorespiratory fitness. To ensure participant safety during the maximum exercise test, all participants underwent a sports medical screening by their general practitioner prior to the study.

### 2.7. Data Analysis

Data analysis was performed in JMP Pro (16.0, SAS Institute Inc., Cary, NC, USA). Prior to conducting statistical analyses, the data were systematically examined to ensure compliance with the assumptions required for parametric tests. For paired *t*-tests, these assumptions included the normality of the differences between paired observations and the absence of significant outliers. Similarly, for repeated measures ANOVA, the assumptions included normality, homogeneity of variances (sphericity), and the absence of outliers. When these assumptions were not satisfied, suitable non-parametric tests were utilized. Specifically, the Wilcoxon signed-rank test was employed as an alternative to paired *t*-tests for comparing PRE–POST and MID–POST differences. For evaluating changes across time points (PRE, MID, and POST), the Friedman test was selected in place of repeated measures ANOVA. Effect sizes were calculated to provide further interpretative value. For parametric tests, standardized mean differences (SMD) using Cohen’s d were computed using the mean differences and pooled standard deviations. When repeated measures ANOVA was appropriate, effect sizes were estimated using partial eta-squared (η^2^). For non-parametric tests, effect sizes were reported as r or W, depending on the specific test used. The r value was calculated as the effect size for the Wilcoxon signed-rank test, while Kendall’s W was used as the effect size measure for the Friedman test. Thresholds for interpretation were as follows: 0.20 for small effects, 0.50 for moderate effects, and ≥0.80 for large effects, as per established guidelines [34,35]. Additionally, post hoc analyses were conducted using Tukey’s Honestly Significant Difference (HSD). These analyses were aimed at assessing the feasibility and clinical efficacy of telerehabilitation HIT. A priori power analysis was not performed due to the nature of the study design. However, guidelines for determining sample size for progression criteria in pragmatic pilot studies were followed, for which a minimum sample size of 10–15 participants is recommended [36].

## 3. Results

Fifteen participants (seven males) were enrolled in the program. Fourteen completed the program successfully without adverse events. One participant discontinued after PRE assessments due to an illness unrelated to CNSLBP. Patient baseline and demographic characteristics are presented in Table 1.

### 3.1. Feasibility Outcomes

System usability (SUS) and subjective experience (IMI) scores ranged from moderate to high at POST. No changes were found from MID to POST for the IMI. Session adherence remained consistently high throughout the trial. The mean session adherence score was 79 out of 100, indicating good overall compliance with the therapeutic program. Motivation and satisfaction scores remained high throughout the intervention phases, but a repeated measures ANOVA revealed motivation decreased (8.0 ± 1.6/10, Δ Difference: −1.2 ± 0.9, *p* = 0.043) over time with a small effect size of 0.19. An overview of feasibility outcomes is provided in Table 2.

### 3.2. Clinical Effectiveness Outcomes

PRE–POST improvements were demonstrated across all measured clinical effectiveness outcomes. Pain decreased (2.7 ± 1.7/10, Δ Difference: −1.8 ± 0.2, *p* = 0.026) with a large effect size of −1.19, and disability scores showed marked improvement (5.8 ± 3.3, Δ Difference: −12.1 ± 10.1, *p* = 0.002) with a large effect size of −0.98. Exercise capacity increased (35.5 ± 10.0, Δ Difference: 4.4 ± 1.6, *p* = 0.048) a moderate effect size of 0.47, while fear-avoidance behavior decreased (35.5 ± 10.0, Δ Difference: −4.4 ± 1.6, *p* = 0.002) with a large effect size of −0.81, reflecting positive changes over time. An overview of the clinical effectiveness outcomes is provided in Table 3.

## 4. Discussion

This clinical trial provides compelling evidence that a technology-supported HIT program administered in a home-based setting is feasible for persons with CNSLBP. The sustained high levels of motivation, satisfaction, and adherence throughout the intervention in the absence of adverse events underscore the safety and acceptability of remotely supervised HIT. Usability assessments further affirmed the functionality of the Physitrack platform. Importantly, this study also demonstrated potentially promising indicators of clinical efficacy, as participants exhibited improvements in physical fitness, reductions in pain intensity, and enhanced functional ability from PRE to POST.

### 4.1. Feasibility

Overall, participants consistently demonstrated high levels of motivation and satisfaction throughout the intervention. Furthermore, an in-depth analysis of the IMI revealed that participants rated the telerehabilitation HIT program equally positive across several dimensions, including interest in target activity, self-efficacy regarding their abilities, perceived effort exerted, feelings of pressure or tension, control over their involvement, and the overall value or usefulness of the program. This outcome substantiates the hypothesis that patients benefit from additional assistance, such as mobile applications like Physitrack, to maintain their exercise regimen and motivation [15,22].

Such support may be particularly critical in HIT, where affective responses can fluctuate significantly due to the demanding nature of the activity. Indeed, higher continuous intensities are generally linked to negative affective states during the activity [37]. However, like in our study, high-intensity interval training (HIIT) appears to deviate from this pattern [38]. In the current study, many participants likewise reported positive affective responses following HIT sessions, potentially counteracting the discomfort experienced during training. This is further confirmed by previous research, suggesting that enjoyment levels during HIIT are comparable to, or even surpass, those of moderate continuous training, indicating that the overall experience of HIIT may be more favorable than traditionally thought [39,40]. These results emphasize the potential of structured interventions using digital health platforms, such as Physitrack, which could advocate to enhance patient engagement and adherence by delivering personalized exercise plans and real-time feedback.

Additionally, this study’s high mean session adherence indicates good therapy compliance with the HIT program. This finding further underscores the effectiveness of the Physitrack application in delivering structured telerehabilitation programs, also evidenced by the favorable SUS scores that highlight the application’s user-friendliness, usability, learnability, consistency, and low complexity. Notably, these results were achieved without direct, in-person supervision, supporting the notion that digital health platforms, such as Physitrack, may effectively bridge the gap typically filled by face-to-face therapist support [41].

While motivation remained high from PRE to POST, it is essential to conduct a critical evaluation of the potential for a decline in motivation over time, especially in the context of extended interventions [42]. Therefore, it is crucial to consider the long-term sustainability of these positive outcomes [43]. Ultimately, motivation and adherence often follow a non-linear pattern, with initial enthusiasm waning over time and leading to a decrease in engagement as the program becomes routine [44]. Thus, the implementation and evaluation of long-term telerehabilitation HIT programs are essential for several reasons: (1) As participants become accustomed to telerehabilitation HIT, the initial sense of challenge and accomplishment may diminish, potentially impacting intrinsic motivation over time [45], (2) As cardiovascular fitness improves, the perceived effort required for HIT sessions may decrease, highlighting the importance of long-term progressions, affecting sense of achievement and consequently motivation [43], or (3) given the potential fluctuations in participants’ personal and professional circumstances, as well as seasonal variations in motivation and adherence, it is essential for long-term studies to account for the effects of these dynamic factors [46].

Although the integration of technology has generally been well received, it may necessitate further optimization to maintain these favorable outcomes. As previous research states, this optimization could involve a more extensive personalization of both the telerehabilitation interventions and the motivational goals and tools provided. Additionally, it is important to address the currently limited possibilities to intervene effectively when motivation drops. A challenge that, while not observed in this sample, is likely to be significant in a clinical setting. This highlights the need for strategies to support adherence over time, which could include exploring technological solutions to provide tailored motivational support and real-time feedback [47]. Addressing these factors is particularly important, as low confidence and diminished motivation are significant barriers to adherence to therapeutic protocols [48,49].

### 4.2. Clinical Effectiveness

In comparison with the findings of an RCT on the effectiveness of HIT as a therapeutic modality in persons with CNSLBP by Verbrugghe et al. (2019), this study also demonstrated significant improvements in critical clinical outcomes. It produced these results with a protocol of only six weeks, compared to twelve [12]. Key metrics such as pain, disability, and exercise capacity improved, with changes exceeding the minimal clinical importance difference (MCID); specifically, pain levels decreased by 1.8 points [50], while disability scores reduced by 12.1% [51], indicating meaningful enhancements in patient functioning and overall well-being. Furthermore, exercise capacity demonstrated a short-term improvement, with an average increase of 4.4 points [52], reinforcing the potential of HIT to enhance physical fitness in this population. Although the MCID for the FACS is not explicitly defined, a significant reduction in maladaptive fear-avoidance beliefs is observed in this study, suggesting a potential positive psychosocial impact of the intervention. These findings, entailing improvement in disability, pain, physical fitness, and fear-avoidance beliefs, further align with outcomes reported in therapist-supported rehabilitation programs and telerehabilitation interventions for persons with CNSLBP [13,19]. Nonetheless, to fully confirm the sustainability and long-term clinical effects of technology-supported training at home, extended follow-up studies are necessary.

### 4.3. Strengths and Weaknesses

A key strength of this trial lies in the exercise therapy protocols, which were rigorously individualized, standardized, and transparently published, thereby enhancing the intervention’s reproducibility. Moreover, the comprehensive measurement and reporting of both feasibility and clinical outcomes underscore the methodological rigor of this pilot study. Notably, the study demonstrated a low drop-out rate of 6.7%, which was further reduced to 0% when excluding cases unrelated to the intervention or CNSLBP. Nevertheless, three important limitations must be acknowledged. First, the open recruitment approach, which excluded patients from hospital-based settings, may introduce selection bias in the study sample. Second, the absence of a control group limits the ability to generalize the findings. Third, the relatively limited sample size represents an additional weakness, as it reduces the statistical power and validity of the results. However, despite these limitations, this pilot clinical trial serves as a valuable stepping stone toward a larger randomized clinical controlled trial, which aims to provide more robust evidence regarding the effectiveness of the intervention [53].

### 4.4. Future Research Directions

Future research should focus on conducting larger randomized controlled trials to confirm the clinical efficacy of a telerehabilitation HIT program for persons with CNSLBP and to enhance the generalizability of the findings. Additionally, extended follow-up studies are necessary to assess the long-term sustainability of improvements in pain, disability, physical fitness, and fear-avoidance behaviors. Furthermore, understanding adherence over time will be essential to improving intervention design, especially for telerehabilitation-based HIT programs. Further studies should also explore the role of digital platforms, like Physitrack, in personalizing HIT programs through tailored motivational support and real-time feedback. These research directions will strengthen the evidence base and enhance HIT’s potential as a sustainable therapeutic option for telerehabilitation.

## 5. Conclusions

A technology-supported HIT program appears to be a feasible and potentially clinically effective intervention for persons with CNSLBP. However, these findings must be interpreted cautiously due to the small sample size and the absence of a control group. Consequently, future research should prioritize evaluating long-term home-based HIT programs tailored to this population. Such studies are essential to validate these interventions’ efficacy and explore their sustained impact on rehabilitation outcomes for persons with CNSLBP.

## Figures and Tables

**Table 1:** Participant characteristics at baseline.

(n = 15)	Mean (SD)	Range
Age (y)	45.9 ± 13.1	[24;63]
Sex (M/F)	7/8	/
BMI (kg/m^2^)	25.8 ± 4.3	[20.8;33.0]
Pain intensity (NPRS, /10)	4.5 ± 1.5	[2;7]
Disability (MODI, %)	17.9 ± 13.7	[4;58]
Exercise capacity (mL/kg/min)	31.1 ± 8.4	[16.2;52.0]

Abbreviations: SD, standard deviation; y, years; M, male; F, female; BMI, body mass index; NPRS, Numeric Pain Rating Scale; MODI, Modified Oswestry Disability Index.

**Table 2:** Feasibility outcomes.

(n = 14)	PRE (SD)	MID (SD)	POST (SD)	Delta	*p*-Value	Cohen’s d/η^2^
Motivation (VAS, /10)	9.2 (0.7)	8.5 (1.1)	8.0 (1.6)	−1.2 (0.9)	0.043 ^a^*	d = 0.26
	/	/	/	/	0.047 ^b^*	η^2^ = 0.19
Satisfaction (VAS, /10)	/	8.8 (1.0)	7.8 (1.8)	−1.0 (0.8)	0.192 ^c^	d = −0.64
System usability (SUS, %)	/	/	91.0 (8.3)	/	/	/
Subjective experience (IMI, %)						
Interest/enjoyment	/	74.1 (7.9)	71.6 (18.5)	−2.5 (10.6)	0.651 ^c^	d = −0.16
Competence	/	61.2 (14.6)	61.9 (16.5)	0.7 (2.1)	0.879 ^c^	d = 0.05
Effort	/	90.9 (7.5)	89.1 (10.7)	−1.8 (3.2)	0.782 ^c^	d = −0.19
Felt pressure	/	29.7 (13.7)	30.5 (9.8)	0.8 (3.9)	0.664 ^c^	d = 0.07
Value/usefulness	/	78.8 (14.6)	71.3 (21.8)	−7.5 (7.2)	0.061 ^c^	d = −0.39
Mean session adherence	/	/	79/100 (8.0)	/	/	

Abbreviations: PRE, baseline measurement; MID, measurement after four in-center sessions; POST, measurement after eight sessions at home; VAS, Visual Analog Scale; SUS, System Usability Scale; IMI, Intrinsic Motivation Inventory; RM ANOVA, Repeated Measures Analysis of Variance; Tukey’s HSD, Tukey’s Honestly Significant Difference. * *p* < 0.05, significant difference between PRE and POST or MID and POST measurements. ^a^ results analyzed using repeated measures ANOVA. ^b^ results analyzed using Tukey’s HSD. ^c^ results analyzed using paired *t*-tests.

**Table 3:** Clinical effectiveness outcomes.

(n = 14)	PRE (SD)	POST (SD)	Delta	*p*-Value	Cohen’s d
Pain (VAS, /10)	4.5 (1.5)	2.7 (1.7)	−1.8 (0.2)	0.026 ^a^*	−1.19
Disability (MODI, %)	17.9 (13.7)	5.8 (3.3)	−12.1 (10.2)	0.002 ^a^*	−0.98
Fear-avoidance (FACS, %)	23.6 (14.3)	13.5 (8.5)	−10.1 (5.8)	0.005 ^a^*	−0.81
Exercise capacity (mL/kg/min)	31.1 (8.4)	35.5 (10.0)	4.4 (1.6)	0.048 ^a^*	0.47

Abbreviations: PRE, baseline measurements; POST, measurement after four in-center sessions; VAS, Visual Analog Scale; MODI, Modified Oswestry Disability Index; FACS, Fear-Avoidance Components Scale. * *p* < 0.05, significant difference between PRE and POST measurements. ᵃ results analyzed using paired *t*-tests.

## Data Availability

The raw data supporting the conclusions of this article will be made available by the authors upon request.
